# Spatial heterogeneity of flesh-cell osmotic potential in sweet cherry affects partitioning of absorbed water

**DOI:** 10.1038/s41438-020-0274-8

**Published:** 2020-04-01

**Authors:** Eckhard Grimm, Daniel Pflugfelder, Jan Hahn, Moritz Jonathan Schmidt, Hendrik Dieckmann, Moritz Knoche

**Affiliations:** 1 Institut für Gartenbauliche Produktionssysteme, Leibniz Universität Hannover, Abteilung Obstbau, Herrenhäuser Straße 2, D-30419 Hannover, Germany; 2 Forschungszentrum Jülich, IBG-2: Pflanzenwissenschaften, Wilhelm-Johnen-Straße, D-52428 Jülich, Germany; 3 Laser Zentrum Hannover e.V., Hollerithallee 8, D-30419 Hannover, Germany

**Keywords:** Plant physiology, Plant cell biology

## Abstract

A fleshy fruit is commonly assumed to resemble a thin-walled pressure vessel containing a homogenous carbohydrate solution. Using sweet cherry (*Prunus avium* L.) as a model system, we investigate how local differences in cell water potential affect H_2_O and D_2_O (heavy water) partitioning. The partitioning of H_2_O and D_2_O was mapped non-destructively using magnetic resonance imaging (MRI). The change in size of mesocarp cells due to water movement was monitored by optical coherence tomography (OCT, non-destructive). Osmotic potential was mapped using micro-osmometry (destructive). Virtual sections through the fruit revealed that the H_2_O distribution followed a net pattern in the outer mesocarp and a radial pattern in the inner mesocarp. These patterns align with the disposition of the vascular bundles. D_2_O uptake through the skin paralleled the acropetal gradient in cell osmotic potential gradient (from less negative to more negative). Cells in the vicinity of a vascular bundle were of more negative osmotic potential than cells more distant from a vascular bundle. OCT revealed net H_2_O uptake was the result of some cells loosing volume and other cells increasing volume. H_2_O and D_2_O partitioning following uptake is non-uniform and related to the spatial heterogeneity in the osmotic potential of mesocarp cells.

## Introduction

In most climates, the leaves, stems and fruits of commercial fruit trees and vines are frequently wetted by rain and/or by dew. However, the fruit skins of many such species are sensitive to wetting, especially when nearing harvest maturity. Examples of such free-water sensitive species are sweet cherries (*Prunus avium*), grapes (*Vitis vinifera*) and plums (*Prunus* *×* *domestica*)^1^.

When the skins of these species are wetted, osmotic water uptake occurs through the wetted portions of the skin. The driving force for the water uptake is the difference in water potential between the adhering water droplet on the outside, and that of the fruit flesh on the inside^2^. The water potential of rain or dew on the outside is usually close to zero (almost no solutes). Meanwhile, the water potential inside the fruit is a composite comprising the summed components of cell/tissue osmotic potential, cell/tissue turgor pressure and cell/tissue matric potential. It is usually considered safe to ignore the matric component of water potential in most mature fleshy-fruit tissues as there are no air spaces (i.e., no gas/liquid interfaces).

Recent studies have established that values of tissue and cell turgor pressure are very low (i.e., almost zero) in mature fleshy fruit so these too are negligible relative to the osmotic potentials of the fruit’s juice^3–7^. This leaves just the osmotic component of fruit water potential as the driving force for water uptake through the wetted skin surface.

In anatomical terms, the tissues accounting for most of the fruit’s juice are the mesocarp and endocarp in berries and the mesocarp in drupes. The flesh accounts for most of the fruit’s fresh mass being large compared to the mass of the skin (thin), and the pit (drupes) or seeds (berries). Anyway, these hard organs do not play a significant role in fruit water relations. The hydraulic architecture just described is common to most, if not all fleshy fruit.

The overlying cuticle is the primary barrier to water movement across the fruit skin^8^. Its resistance to water movement is higher, by several orders of magnitude, than that of cell walls and plasma membranes^9^. This is considered to imply that the tissues enclosed by the cuticle are likely to be in close water potential equilibrium and, thus, that the fruit represents a thin-walled pressure vessel filled with a sugary solution. This model is often used when analysing fruit water relations, fruit cracking^10,11^ or the stress-strain distributions in the fruit skin^12^. However, this model contains some implicit assumptions that have not always been given adequate consideration—hence it may not be valid.

First, recent studies have established that epidermal cells of sweet cherry fruit plasmolyse when exposed to juice from the same fruit. This unexpected behaviour indicates the osmotic potential of the exocarp is less negative than that of the mesocarp—the expressed juice from a cherry is predominantly from the mesocarp^13^. More surprisingly, the osmotic potential difference between exocarp and mesocarp is remarkably stable with time; for example, the difference does not decline when the fruit are stored for quite lengthy periods^14^. Moreover, the magnitude of the difference does not depend on the transpiration history of the fruit^11^.

Second, as the fruit approaches maturity, all import of vascular water and carbohydrate to the fruit is via the phloem^15^. In sweet cherry, the arrangement of the vasculature within the fruit is not uniform^16^. Fruit vasculature comprises five major bundles. Three bundles supply the mesocarp and two supply the ovules. The major bundles branch into radial veins in the inner mesocarp. In the outer mesocarp these veins form a net of minor veins that interconnect via numerous anastomoses. The exocarp is devoid of minor veins^16^.

Third, fruit cracking may be induced by localised exposure of the fruit surface to water. If only a part of the fruit surface is wetted (with the non-wetted areas continuing to transpire), the wetted surface will crack, even though the fruit may suffer a net loss of water—i.e., the amounts of water transpiring through the non-wetted areas, exceeding those taken up osmotically through the wetted areas^17,18^.

Fourth, the amount of water triggering fruit cracking is very much lower when uptake occurs through the fruit surface, compared to if water is perfused into the flesh through a hypodermic needle^18^.

Fifth, there is an acropetal gradient in water potential within a sweet cherry from the less-negative pedicel region to the more-negative stylar scar region^11^.

All these observations are difficult to explain in terms of the conceptual model of the sweet cherry as a thin-walled pressure vessel, filled with a homogenous osmotic solution. Instead, a model that involves a persistent heterogeneity in water potential more easily explains most (or all) of these behaviours.

Using sweet cherry as the model system, the objectives of this study, were to map (1) the partitioning of water (H_2_O) and heavy water (D_2_O) following osmotic uptake through the skin, with resolution at the level of individual tissues and individual cells, and to map (2) the distribution of osmotic potential within the fruit. We used magnetic resonance imaging (MRI) and optical computer tomography (OCT) to visualise D_2_O and H_2_O partitioning and the resulting change in cell volumes non-destructively. In MRI, D_2_O is a ‘negative’ tracer. When D_2_O replaces H_2_O, it reduces the H_2_O signal following submersion and uptake.

## Results

**Fig. 1:**
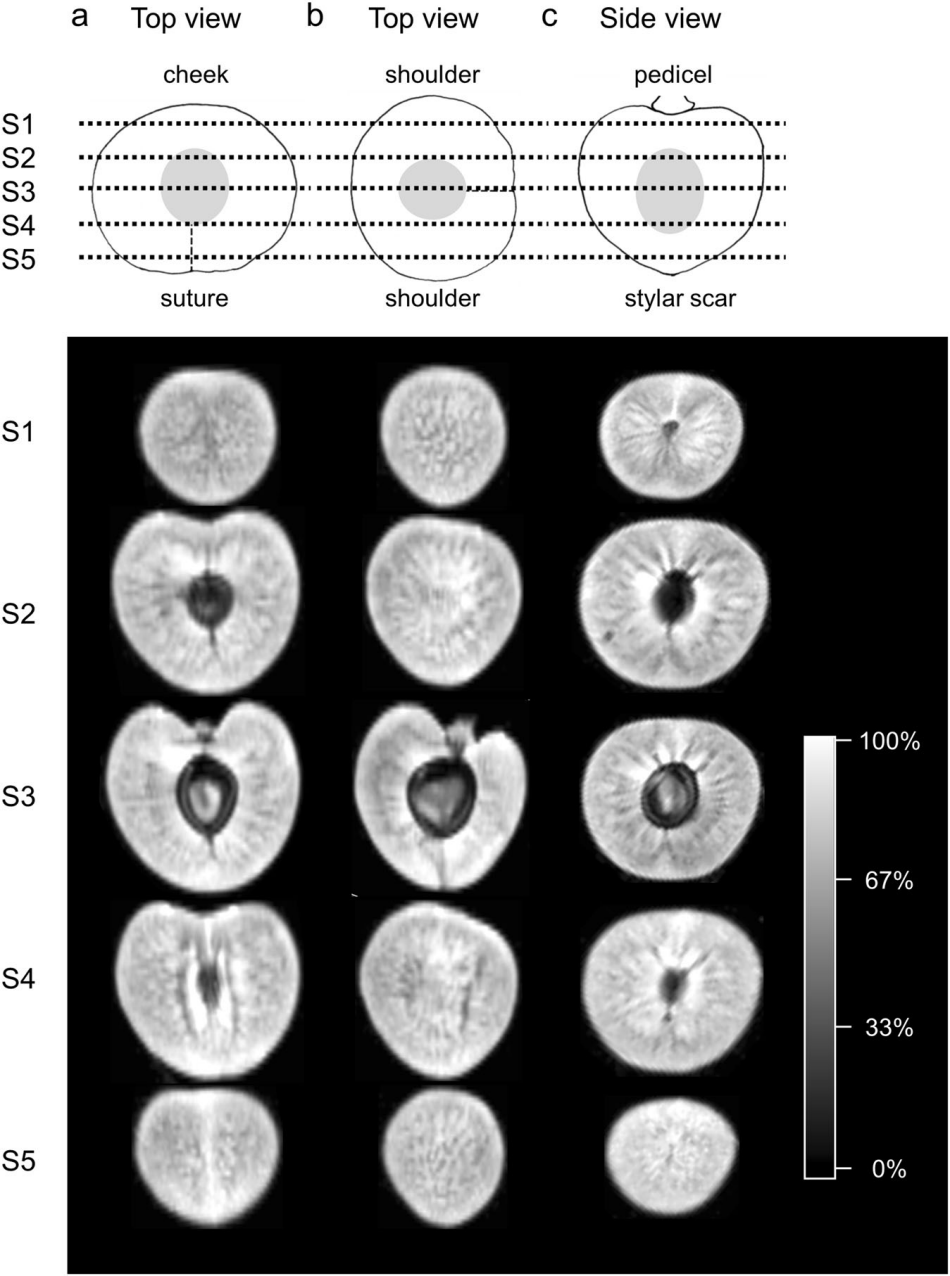
Water partitioning in a mature sweet cherry as determined by magnetic resonance imaging. Columns (**a**–**c**) represent different planes of virtual sequential sections S1 to S5 through the fruit as indicated by the dashed lines. Longitudinal sections in the plane of the two shoulders (column **a**), in the plane of cheek and suture (column **b**), and latitudinal (equatorial) sections normal to the pedicel stylar scar axis (column **c**)

Sequential longitudinal and latitudinal virtual sections through fruit revealed considerable heterogeneity in the partitioning of H_2_O (Fig. 1). A net type pattern dominated in the first and last tangential sections through the outer mesocarp (Fig. 1a, b, sections S1, S5). As consecutive subsequent sections approached the plane of the endocarp in the fruit, a pattern of radial channels was detectable in the inner mesocarp, most clearly in the two shoulders left and right of the endocarp (Fig. 1a, c, S3). The major median and the lateral bundles were detected by MRI (median bundle in Fig. 1a, section S2; lateral bundles in Fig. 1a, section S4). In addition, the vasculature and the intercellular space in the pedicel cavity were also resolved by MRI (Fig. 1c, section S1).

**Fig. 2:**
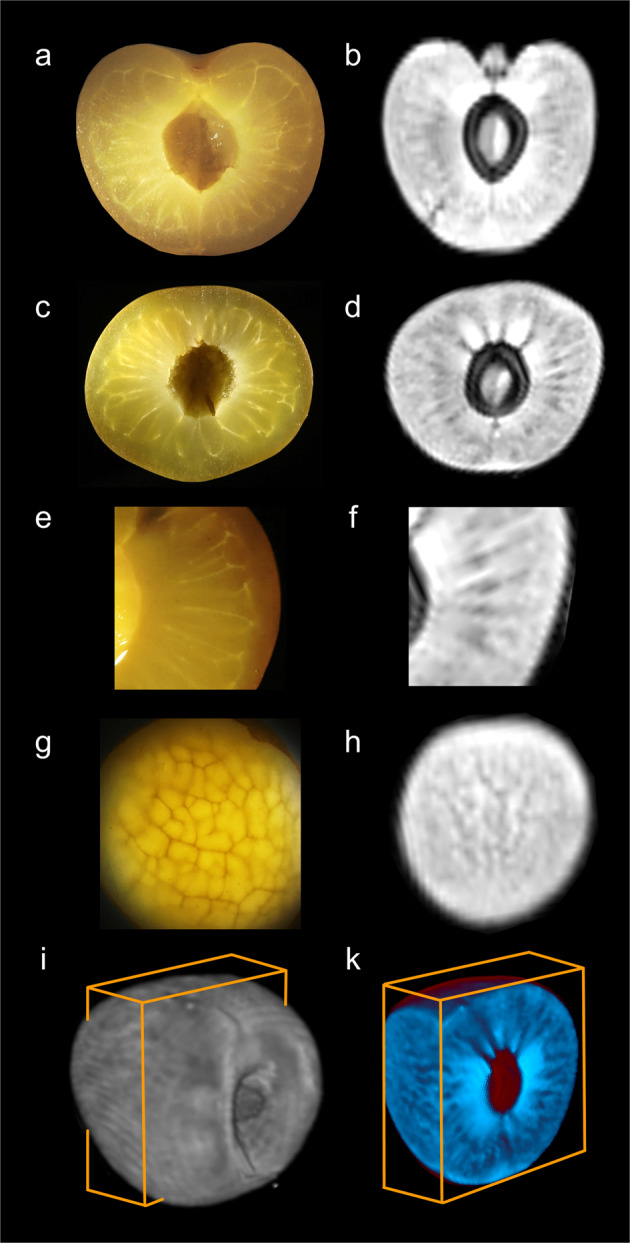
Anatomy and H_2_O partitioning in mature sweet cherry fruit. Light micrographs (**a, c, e, g**) of longitudinal (**a**) and equatorial (**c, e**) cross sections through whole fruit (**a, c**) or shoulder region (**e**) revealing vasculature of veins. Most of the veins extended radially through the inner mesocarp. Light micrograph of tangential section through the outer mesocarp demonstrating net of minor veins (**g**). Magnetic resonance imaging (MRI) of H_2_O partitioning (**b, d, f, h**; for scaling see Fig. 1) in a virtual slice of 1 mm thickness in the planes corresponding to the microscopic sections shown in **a, c, e, g**. 3D-MRI images of H_2_O partitioning (**i**, **k**). Whole fruit with box indicating planes of virtual sections (**i**). Virtual section (**k**) revealing a net type pattern of H_2_O partitioning in the tangential plane at the front face of the box and a radial pattern of veins at the side face. For sketch and nomenclature of the different regions see Fig. 1

The heterogeneous pattern of the H_2_O distribution was not random, but closely related to the distribution of the vasculature within the fruit (Fig. 2). The periphery of the fruit comprised the exocarp and was totally devoid of bundles and veins (Fig. 2a, c, e). The outer mesocarp was characterised by a net of minor veins that was oriented predominantly tangentially to the surface (Fig. 2e, g). In the inner mesocarp a radial pattern of veins was observed, there were no veins immediately adjacent to the endocarp. The MRI signal intensity reflected the H_2_O partitioning in the mesocarp and essentially mirrored the distribution of the vasculature within the fruit (Fig. 2b, d, f, h): A net type pattern parallel to the surface in the outer and a radial pattern in the inner mesocarp (Fig. 2f, h, k).

**Fig. 3:**
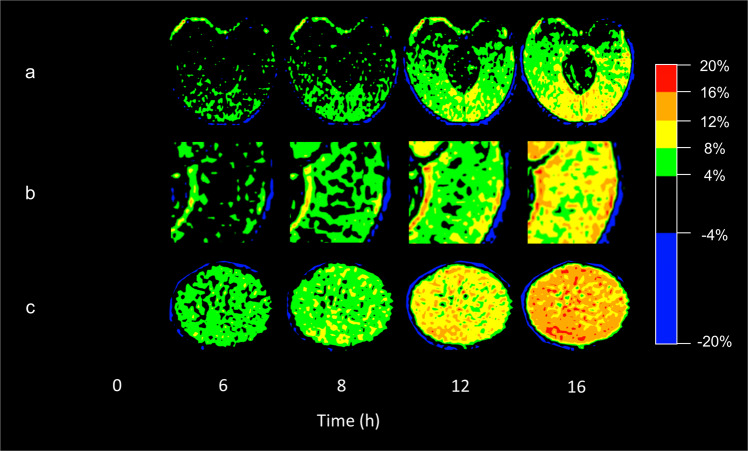
Time course of D_2_O uptake into sweet cherry fruit as viewed by magnetic resonance imaging. Virtual sections through whole fruit (**a**), detailed view (**b, c**) of equatorial cross section through the shoulder (**b**) and of tangential section through the stylar scar region (**c**). For sketch and nomenclature of the different regions see Fig. 1, (**a, b, c**) correspond to (**a**-S3), (**c**-S3), (**c**-S5) in Fig. 1, respectively. The D_2_O label increases as colour changes from black to green to yellow and red

D_2_O uptake was highest in the stylar scar region, followed by the shoulder (Fig. 3a). The lack of a uniform H_2_O signal reduction by D_2_O indicated heterogeneity also in the partitioning of D_2_O. Areas of marked D_2_O accumulation were located adjacent to areas without D_2_O accumulation (Fig. 3b, c). A radial pattern of D_2_O partitioning emerged with time in the inner mesocarp (Fig. 3b), whereas a net type pattern was observed in the outer mesocarp (Fig. 3c).

**Fig. 4:**
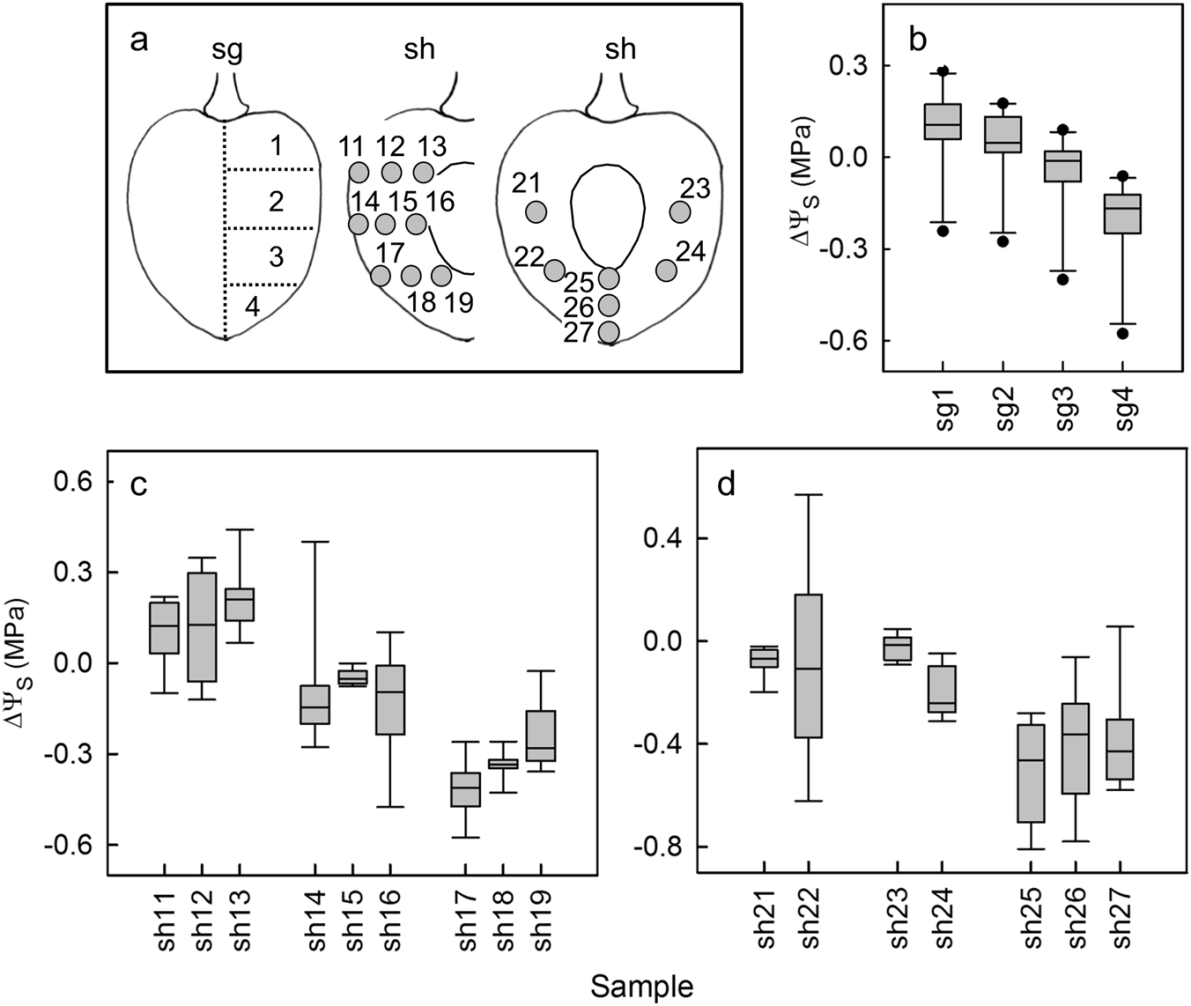
Mapping of the osmotic potential (Ψ_*s*_) in different regions of a mature sweet cherry fruit. Sketch of regions that were sampled (**a**). Osmotic potentials were expressed as the difference of the osmotic potential (ΔΨ_*s*_) calculated by subtracting the Ψ_*s*_ at the respective site from the mean Ψ_*s*_ of the whole fruit. Osmotic potentials of latitudinal sections perpendicular to the stylar scar/pedicel axis (**b**); osmotic potentials in the outer and inner mesocarp (median position and close to endocarp) in latitudinal sections in different planes of the shoulder region (**c**); osmotic potentials of inner mesocarp in different planes of the shoulder and in the outer and inner mesocarp (median and close to the pit) of the stylar scar region (**d**). For nomenclature of the different regions see Fig. 1

The osmotic potential of the juice within the fruit was not spatially constant. An acropetal gradient of decreasing osmotic potential was detected in the fruit from the pedicel cavity (less negative) towards the stylar scar region (more negative) (Fig. 4a–d). There were no consistent differences between osmotic potential values in the outer or the inner mesocarp adjacent to the pit neither in the equatorial plane (Fig. 4a, c), nor in the stylar scar region (Fig. 4a, d).

**Fig. 5:**
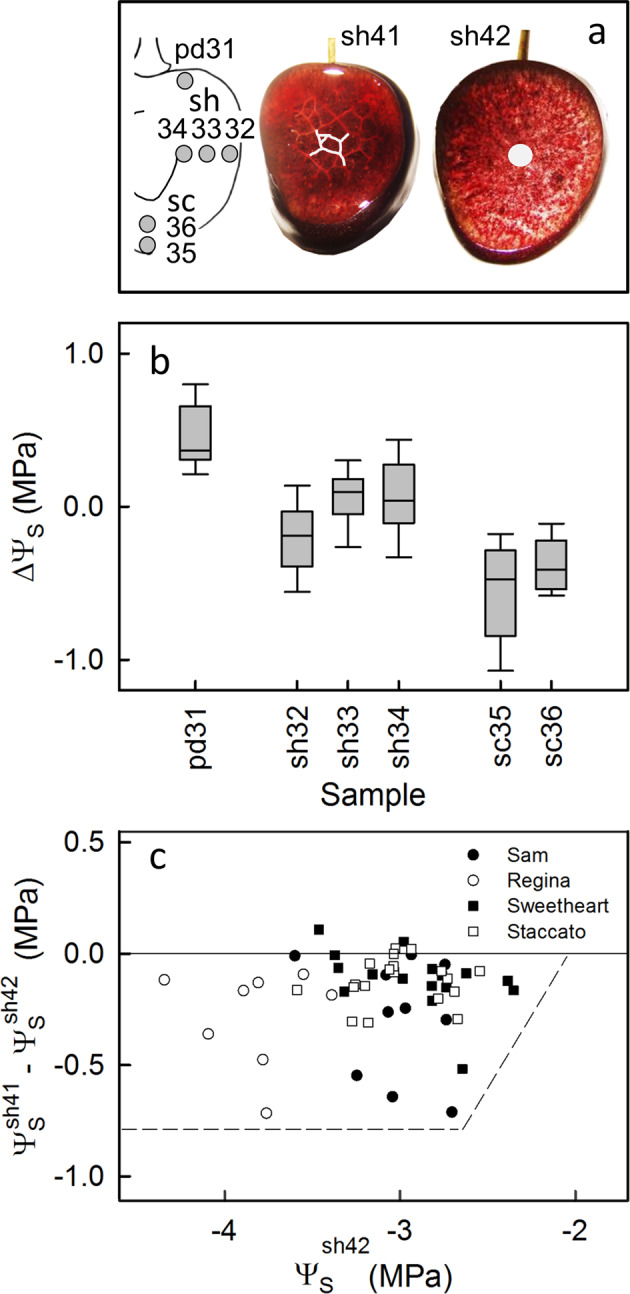
Mapping of osmotic potentials (Ψ_*s*_) in different regions of a mature sweet cherry fruit relative to the vascular system. Sketch of fruit indicating different sampling sites (**a**). The photographs indicate the sampling site at the minor vein net of the outer mesocarp (**a**, sh2) and that between the radial veins in the inner mesocarp (a, sh3). Difference in osmotic potential (ΔΨ_*s*_) between the Ψ_*s*_ at the site of sampling (see sketch in **a**) minus the mean Ψ_*s*_ of the respective whole fruit; pd31 = pedicel cavity, sh = shoulder, sh32 = outer mesocarp with minor vein net, sh33 = inner mesocarp between radial veins, sh34 = inner mesocarp close to the pit, sc = stylar scar region, sc35 = outer mesocarp with minor vein network, sc36 = inner mesocarp with radial veins in stylar scar region (**b**); difference in osmotic potentials between the minor vein net of the outer mesocarp (sh41) and the radial veins of the inner mesocarp (sh42) plotted against the osmotic potential of the inner mesocarp (sh42). Data represent four different sweet cherry fruit cultivars (**c**)

Further analysis of the spatial distribution of osmotic potential revealed more negative osmotic potentials in the vicinity of the minor vein network in the outer mesocarp, particularly in the cheek region, compared to in the inner mesocarp (Fig. 5a, b). The variability in osmotic potential was high, but the difference in osmotic potential in the minor vein region of the outer mesocarp relative to that in the inner mesocarp with radial veins was consistent across all four cultivars (Fig. 5c). The maximum difference detected across all samples was 0.7 MPa.

**Fig. 6:**
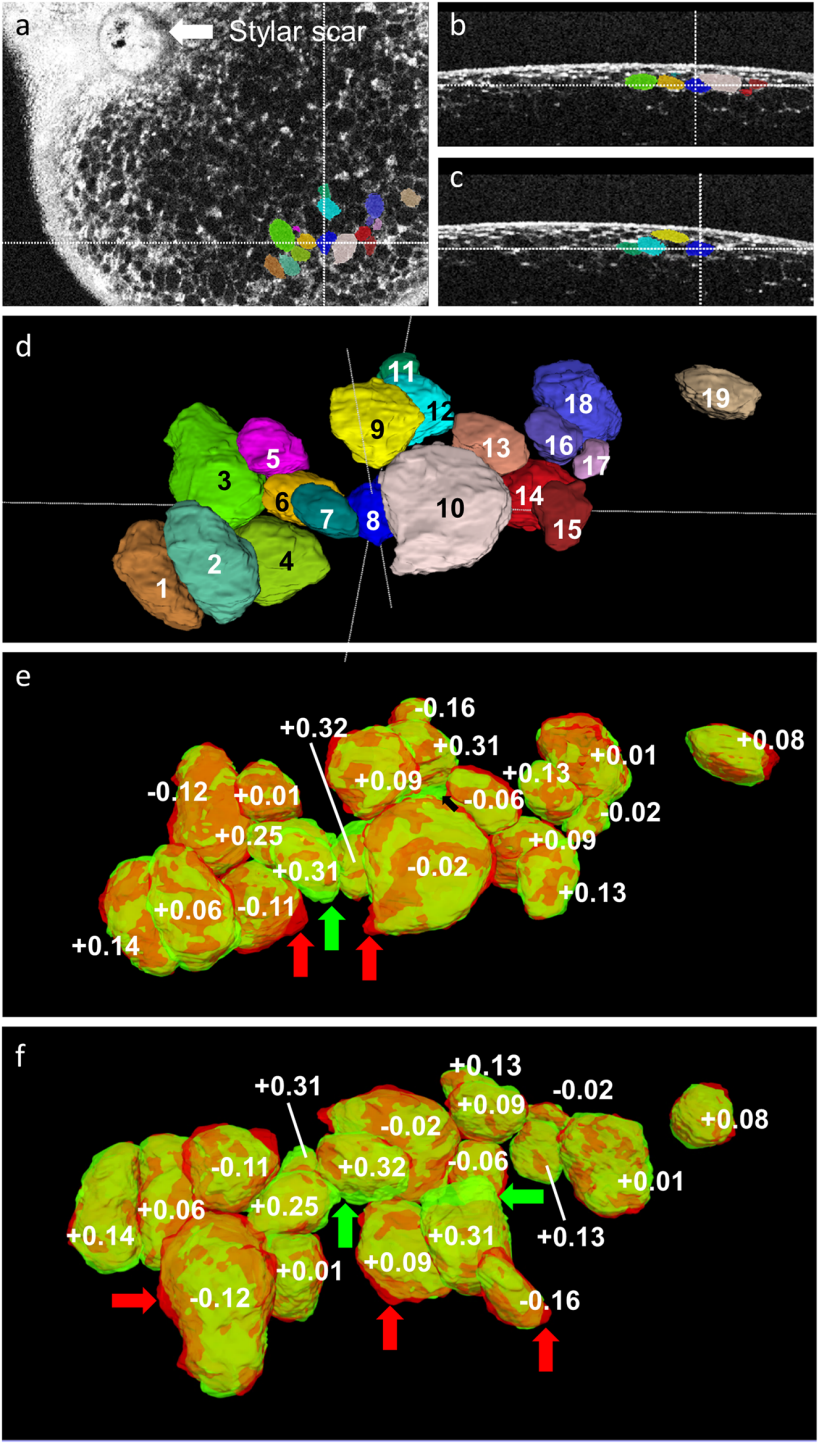
Optical coherence tomography images of cells in the outer mesocarp in the stylar scar region of sweet cherry fruit incubated in water. Overview of stylar scar region (**a**); virtual cross sections showing position of cells relative to the fruit surface (**b, c**); **c**: same view as (**b**), but rotated by 90°; **d**: same cells as in **b**, **c** at higher magnification. The change in volume of these cells was monitored during incubation in water; **e**: Volume changes of the same cells as (**d**) during 6 h of incubation in deionised water; **f**: same cells as (**e**), but image tilted by 180°. Numbers indicate relative changes in volume. Positive signs indicate volume increase of cell, negative signs volume decrease of cell. Green arrows point to green areas of the surface of cells that indicate expanding regions of a cell. Red arrows point to red areas depicting shrinking regions of a cell. Brown areas identify regions of cells that do not change in area

OCT measurements allowed non-destructive monitoring of volume change of individual cells in the outer mesocarp, over time (Fig. 6). Cells in the outer mesocarp were selected that had clear cell walls throughout a 6 h time course of submersion. For some cells, the volume decreased by up to 15%, while in others, the volume remained constant or increased by up to 30%. Cells that exhibited large increases in volume were grouped closely together (cells 6, 7 and 8 in Fig. 6e, f). It is interesting that (1) the increase in volume of a cell was not uniform in all directions but focused in specific regions of the cell, (2) the expanding regions of neighbouring cells were typically oriented towards each other and (3) there was no detectable formation of cracks in the skin within the 6 h incubation period and hence, no direct spatial relationship between the penetration of water through the skin and the distribution of the water taken up.

The change in volume of a cell was not correlated with the original size of the cell at the onset of the experiment (*r* = −0.44, *n* = 19, *P* = 0.06; Supplementary Fig. 1).

## Discussion

Results presented herein establish (1) a preferential accumulation of water in a net-type structures in the outer mesocarp and radial channels in the inner mesocarp of mature sweet cherry fruit. These distribution patterns reflect the more negative osmotic potentials in close proximity to the vascular system, (2) a longitudinal gradient of increasing water uptake rate and thus decreasing (more negative) osmotic potential from the pedicel cavity region to the stylar scar region and (3) water uptake by individual cells can differ markedly between neighbouring cells and over short distances, with some cells showing considerable water loss, while others close by showing marked water uptake. Our findings indicate considerable heterogeneity in the partitioning of water following uptake through the skin. This heterogeneity occurs at the tissue level (systematic) and also at the cell level (apparently random).

## Tissue level heterogeneity

The distribution of H_2_O and D_2_O within the fruit revealed a consistent heterogeneous pattern. The appearance of both the net and the rays indicates regions of preferential H_2_O/D_2_O distribution, with the interstitial regions containing less H_2_O/D_2_O. Consistent with the pattern of H_2_O accumulation was the finding of a decrease in osmotic potential (more negative) along the radial veins and in the minor vein network of the vascular system. The interstitial regions, in which H_2_O/D_2_O accumulation was less or absent, suggests the osmotic potential was less negative. These cells were less attractive to water. These observations are mechanistically related. Due to a very low cell turgor, the osmotic potential difference is the sole force driving both the magnitude and the direction of diffusion of water. Hence, the pattern of cell osmotic potential in the fruit determines the pattern of H_2_O/D_2_O distribution in these experiments. Thus, any water taken up by the fruit will accumulate preferentially in tissues and in cells possessing more negative osmotic potential. This would seem to be the case for tissues and cells finding themselves in close proximity to the radial and minor veins.

While these experiments satisfactorily establish that cells finding themselves close to the veins are of more negative osmotic potential, they offer no mechanistic explanation for this observation. We offer the following possible explanations: (1) There is ongoing leakage/diffusion of solutes out of the vascular bundles into the cells of the interstitial tissues—in particular out of the minor vein net, but there seems to be a failure for these lost solutes to be loaded back into the vascular bundles and veins; and (2) There would seem to be limited movement (diffusion? other?) of the leaked solutes through the interstitial tissues, so as to render tissue and cell osmotic concentrations less heterogeneous.

## Cell level heterogeneity

Our three-dimensional OCT data, allow volume changes of individual cells of the outer mesocarp to be monitored during incubation in water. To minimise heterogeneity of water uptake, our analyses were restricted to fruit that remained intact (i.e., uncracked) during the entire incubation period of up to 6 h. Our results reveal that the net fruit mass increase observed during incubation in water (for review see ref. ^8^) is the effect of some cells loosing water and others taking up water. Sometimes, a cell losing water was found to be immediately adjacent to one taking up water. We recorded individual cell-volume increases of as much as 30%.

Furthermore, for a particular cell, the change in volume was not uniform, with some parts of the cell wall increasing in surface area, and thus in enclosed volume, and others decreasing. In other words, cell shape changed markedly. Interestingly, when immediately adjacent cells were examined, those areas of their cell walls that were touching suffered similar patterns of extension. We do not understand the trigger nor the mechanism responsible for this coordinated, localised response of neighbouring cells. We offer the following comments: (1) The localised response of a number of adjacent cells may be a result of coordinated cell leakage or bursting. For example, if an individual cell began to leak/burst, its cytoplasmic contents would be released into the cell wall free space and into any fluid-filled intercellular spaces. As determined from the juice expressed from sweet cherry mesocarp tissue, the symplast of these cells contains high concentrations of malic acid (70 mM^19^) and is very acidic (pH 4.2^20^). Malic acid is known to decrease the modulus of elasticity of the cell wall material, possibly by desorbing and complexing cell-wall bound Ca^20,21^. The effect of symplastic leakage would remain localised if diffusion of malic acid in the apoplast was slow. Slowed diffusion is expected where one or several of the following conditions are met: (1) The cross-sectional area of the diffusion path in the cell wall free space is small—e.g., in the thin wall of a parenchyma cell. (2) The diffusion path is tortuous. (3) If there is some barrier to diffusion of water such as a swollen cell wall that restricted convective flow resulting from osmotic water uptake. Cell wall swelling is likely to occur in mature sweet cherry fruit particularly when turgor is low and cells plasmolyse^13,22^. (4) Cell:cell variability in osmotic potential would contribute to differences in water uptake between cells. Such variability in epidermal cell osmotic potential has been observed in sweet cherry^13^. In that study, a decrease in the osmotic potential (more negative) of the incubation solution, increased that fraction of epidermal cells suffering plasmolysis. The range between the maximum and minimum osmotic potentials (i.e., where the first and the last epidermal cells plasmolysed) was surprisingly wide, >1 MPa^13^. This indicates that the water potentials of the population of epidermal cells differed by this amount. Incidentally, the water potential difference between the skin (less negative) and the flesh (more negative) represents the driving force for an internal re-distribution of water from skin to flesh that occurs postharvest. This redistribution of water causes the ‘orange peel’ disorder in sweet cherry^14^. Also, a major fraction of fruit cracks in the stylar scar region which is consistent with the more negative osmotic potential measured in this study^23,24^.

Just how such large differences in water potential can occur over such short distances is unknown. Possibilities include: (1) In the outer mesocarp, differential unloading from the minor vein network of carbohydrates into the apoplast and/or (2) differential uptake from the apoplast into the symplast^25^ and/or (3) differential transport to more distant groups of cells. This question merits further study.

## Conclusion

The popular view is that a fleshy fruit behaves like a thin-walled pressure vessel, filled with a sugary solution, which is of uniform composition and concentration. This model should probably now be discarded in the light of new information. Instead, a mature fruit is not at static equilibrium with respect to its osmotic potential and water potential. A fleshy fruit is more realistically approximated by a system in transient steady state, where gradients in osmotic potential and water potential arise from continuing influxes of carbohydrates to the fruit. Because the osmotic influx is likely variable on a temporal scale (as a result of diurnal changes in light intensity and temperature etc.) and also variable on a spatial scale (as a result of the uneven arrangement of the vascular system), a true equilibrium of osmotic potential and water potential is unlikely ever to be reached. This introduces a highly variable element to all processes relating to the uptake and distribution of water associated with rain cracking of fruit. This makes systematic assessment difficult. Although the above findings were made using sweet cherry as the experimental subject, we would expect them to be applicable to other fleshy fruit that accumulate large amounts of osmotically active carbohydrates during growth and ripening.

## Materials and methods

### Plant material

Mature sweet cherry fruit (*Prunus avium* L.) (cvs. ‘Dönissens Gelbe’, ‘Gil Peck’ ‘Regina’, ‘Sam’, ‘Schneiders Späte Knorpelkirsche’) were sampled from trees cultivated in a greenhouse or under a rain shelter at the Horticultural research station in Ruthe (52.2 N, 9.8E) or an experimental orchard in Hannover (52.4 N, 9.7E). All trees were grafted on ‘Gisela 5’ rootstocks (*P. cerasus* L. × *P. canescens* Bois) and cultivated according to current regulations for integrated fruit production. Fruit for MRI were harvested in Ruthe, held under non-transpiring conditions at 2 °C, transported to the Jülich laboratory and processed on the same day. Fruit from the orchard in Hannover was used for quantifying osmotic potential. For the experiments, we selected uniform, mature fruit that were free of visible defects.

### Anatomy

Microscopic studies were carried out using ‘Gil Peck’ fruit fixed in Karnovsky solution^26^. The cultivar was selected because of the lack of anthocyanins that would interfere with light microscopy. Hand sections were prepared using a razor blade. Specimens of 7 fruits were inspected under transmitted and incident (reflected) light microscopically (microscope MZ6; Leica, Wetzlar, Germany) or macroscopically. Digital images were taken (camera DP71; Olympus, Hamburg, Germany and EOS 550D, lens EF-S 60 mm f/2.8 Macro USM; Canon, Tokyo, Japan) and processed using image analysis software (cellSens Dimension 1.7.1; Olympus).

### Magnetic resonance imaging

Mature ʹSamʹ fruit were sampled, the pedicel was cut close to the receptacle. The pedicel cavity was sealed using epoxy adhesive (Uhu plus schnellfest, Uhu GmbH and Co. KG, Bühl, Germany). Fruit were subsequently mounted in a custom holder. The atmosphere inside the holder was maintained at high humidity to minimise transpiration. For analysing uptake and distribution of heavy water (D_2_O) fruit was incubated in D_2_O (Sigma-Aldrich, Taufkirchen, Germany). To prevent fruit cracking the D_2_O solution contained CaCl_2_ at 10 mM. MRI scans were conducted using 8 (H_2_O) and 4 fruit (D_2_O), respectively.

Imaging was done in a 4.7 T magnet (Magnex, Oxford, UK) equipped with a MR Solution console (MR Solutions, Guildford, UK) (for details, see ref. ^27^), using a birdcage radio frequency (RF) coil of 100 mm inner diameter (Varian). The sample holder was positioned by an industrial pick-and-place robot (Mini-Liner 3.0 Alu; Geiger Handling, Dornhan, Germany) mounted on top of the magnet. This setup allowed repeated observations of the same fruit such that time courses of uptake and distribution of D_2_O could be established on an individual fruit basis. The following settings were used: Spin-Echo Multi-Slice Multi-Echo sequence, slice resolution = 0.5 × 0.5 mm^2^, field of view = 96 × 96 mm^2^, slice thickness = 0.7 mm, 110 slices, TR = 14000 ms, TE = 15 ms, echo spacing = 15 ms, 5 echoes, spectral width = 50 kHz, acquisition time per cycle ~45 min. These parameters allowed monitoring four fruit simultaneously. Water content and T_2_ values were determined by a pixel-wise exponential fit. Absolute water content values calibration was established using reference probes of 5 mM Ni(NO_3_)_2_ within the field of view.

MRI scans were processed using image analysis software (MeVisLab 2.6.1.; MeVis, Bremen, Germany). For virtual cross sections fruit were cut into slices of 1 mm thickness. The D_2_O taken up reduces the signal. The D_2_O distribution in virtual sections was thus calculated by subtracting the measured water content obtained on a section at any one time from ‘feeding’, from the background signal for the same fruit, for the same section, at time zero. The differential signals were expressed as heat maps with black indicating ‘no’ D_2_O accumulation and red indicating ‘high’ D_2_O accumulation. Negative differences are indicated in blue.

### Osmotic potential

Fruit were processed immediately following harvest or held at 2 °C for a maximum of 36 h. Fruit were sectioned longitudinally along the pedicel/stylar scar axis in the cheek/suture plane to obtain two symmetrical halves. The pit was removed and the juice expressed mechanically from one of the two halves by crushing. The other half was cut normal to the pedicel/stylar scar axis into four sections of approximately equal thickness from which the juice was extracted. Aliquots (10 µl) of juice were analysed by water vapour pressure osmometry (Vapro 5520 and 5600; Wescor, Logan, UT, USA). The number of replicates was 10. To obtain a higher spatial resolution of the distribution of osmotic potential, the procedure was modified in subsequent experiments. For this, juice was extracted under a stereomicroscope using disposable 0.5 or 1 ml micro syringes. The needle was inserted in the tissue in a preselected region and the juice taken up into a glass capillary (inner and outer diameters 0.58 and 1 mm, length 150 mm; Hilgenberg, Malsfeld, Germany). The juice in the capillary was analysed by water vapour pressure osmometry within 1 h of collection. The sample volume for high-resolution measurement was reduced to 2 µl. The number of replicates was 8. Preliminary experiments were conducted to optimise handling of these small volumes of juice without significant artefactual changes in concentration or amount.

### Optical coherence tomography

Fruit of mature ‘Dönissens Gelbe’ sweet cherry were inspected for freedom of visual defects in the stylar scar region. The pedicel was cut flush with the receptacle. The cut end of the pedicel, the pedicel and the pedicel cavity were sealed using silicone rubber (SE 9186; Dow Corning, Midland, MI, USA) to prevent water uptake and cracking in this region. Sets of 10 fruit were mounted in a custom water bath under the OCT lens such that the distance between the OCT lens and the stylar scar region was constant for all fruit.

The OCT system used was as described previously^28^. Briefly, the OCT comprised a lens (LSM03-BB; Thorlabs, NJ, USA), a pair of galvanometer scanning mirrors (6210 H; Cambridge Technology, MA, USA), a superluminescent diode having a central wave length of 835 nm (BLMS‐mini‐351‐HP2‐SM‐I; Superlum Diodes, Ireland) and a long-range spectrometer (Cobra UDC, Wasatch Photonics, NC, USA). Image acquisition and processing were carried out using the custom laboratory framework smartLab (Laser Zentrum Hannover e.V., Germany). Settings for measurements were: 500 B-scans per fruit, a maximum depth of penetration into the fruit of ca. 500 µm, a field of view of 500 × 500 × 2048 voxels corresponding to 9370 × 9370 × 8400 µm³ in air, recording time for a full 3D image set <1 min per fruit. A total of 3 × 10 fruit was scanned.

The experiment was initiated by filling the bath with deionised, degassed water thereby submerging all fruit simultaneously. The stylar scar region of each fruit was scanned individually, before the next cycle was initiated.

Data were analysed as described previously^28^. Regions of interest (ROI) were selected in a location of the fruit that: (1) remained free of visible cracks throughout the 6 h incubation period and where (2) the cell walls were clearly visible in all planes. Images of ROIs were processed by magnifying and filtering. Volumes of 19 cells were quantified by segmenting starting from the centre of a cell. Corrections for missing data were applied, where needed. Cell volumes and changes relative to those when incubation was initiation were calculated. Voxels were converted to metric volumes using the following conversions: The ROIs had 1000 × 1000 × 500 voxels where 1 voxel corresponded to a volume of 271.1 µm³. Using this setup, the change in volume of individual cells of the outer mesocarp was monitored.

### Data presentation and statistics

All images of fruit are representative for the population of fruit investigated. Unless individual data points are shown, data in line graphs are presented as means ± standard errors. In boxplots the upper and lower ends of the box represent the 25 and 75 percentiles, respectively, and the horizontal line in the box, the median. The bars indicate the 10 and 90 percentiles, the plotted data symbols represent outliers. Data were analysed by analysis of variance and regression analysis using the statistical software package SAS (version 9.1.3; SAS Institute Inc., Cary, N.C.).

## Supplementary information

Supplementary Figure 1

Supplementary Figure 1

Data Set 1

## Data Availability

The data that support the findings of this study are available from the corresponding author upon reasonable request.
