# Supplementary material for: Spatial heterogeneity of flesh-cell osmotic potential in sweet cherry affects partitioning of absorbed water
Source: Hortic Res. 2020 Apr 1;7:51. doi: 10.1038/s41438-020-0274-8 (PMC7109129; doi:10.1038/s41438-020-0274-8)

**Supplementary Fig. 1** **Relationship between absolute (a) or relative change in volume of cells (b) and the initial size of the cells during a 6 h incubation period in deionised water.** Data were taken from Fig. 6.


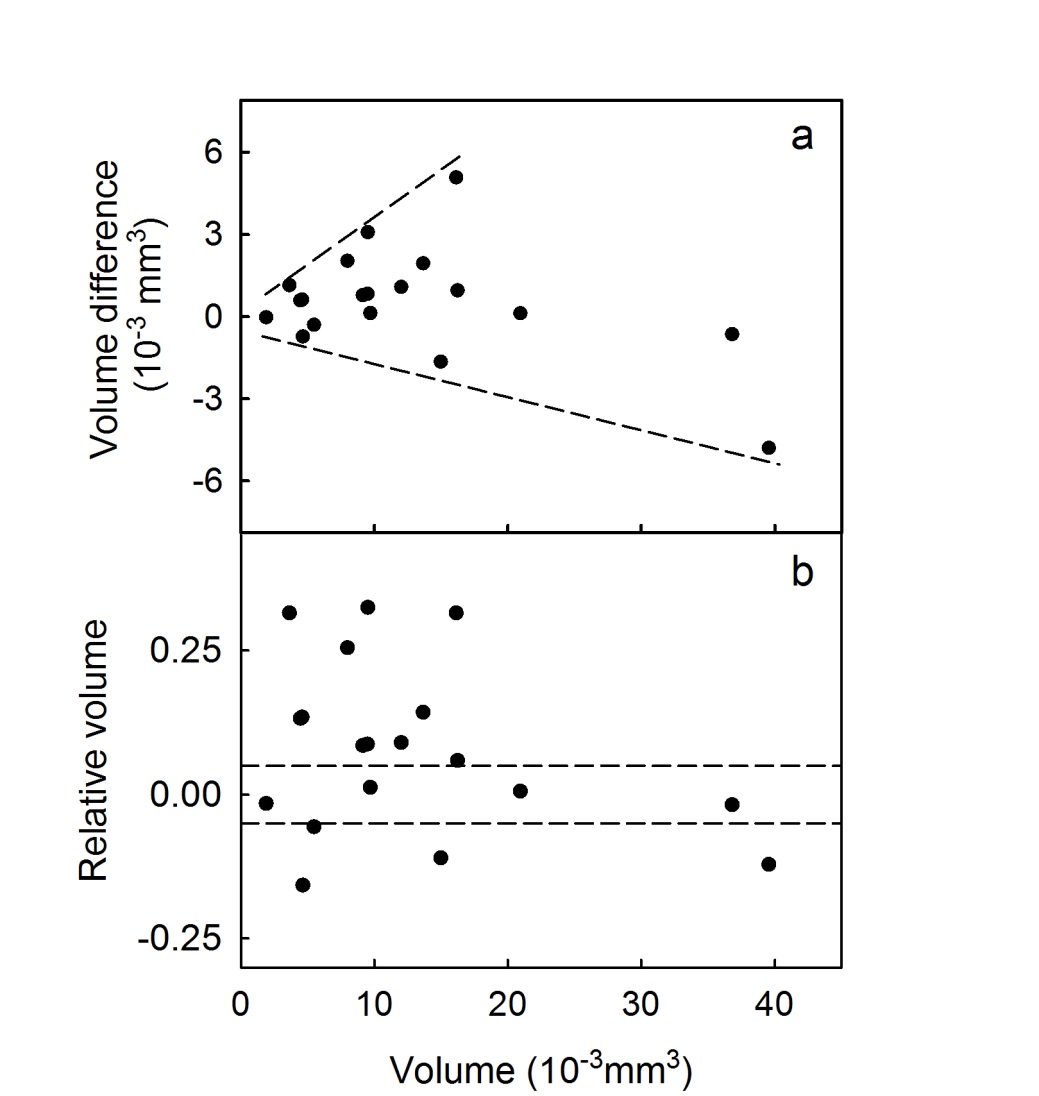

Supplement: Supplementary file 1 — Supplementary Figure 1 [file 41438_2020_274_MOESM1_ESM.docx]
