# Supplementary figures and images for: Spatial heterogeneity of flesh-cell osmotic potential in sweet cherry affects partitioning of absorbed water
Source: Hortic Res. 2020 Apr 1;7:51. doi: 10.1038/s41438-020-0274-8 (PMC7109129; doi:10.1038/s41438-020-0274-8)

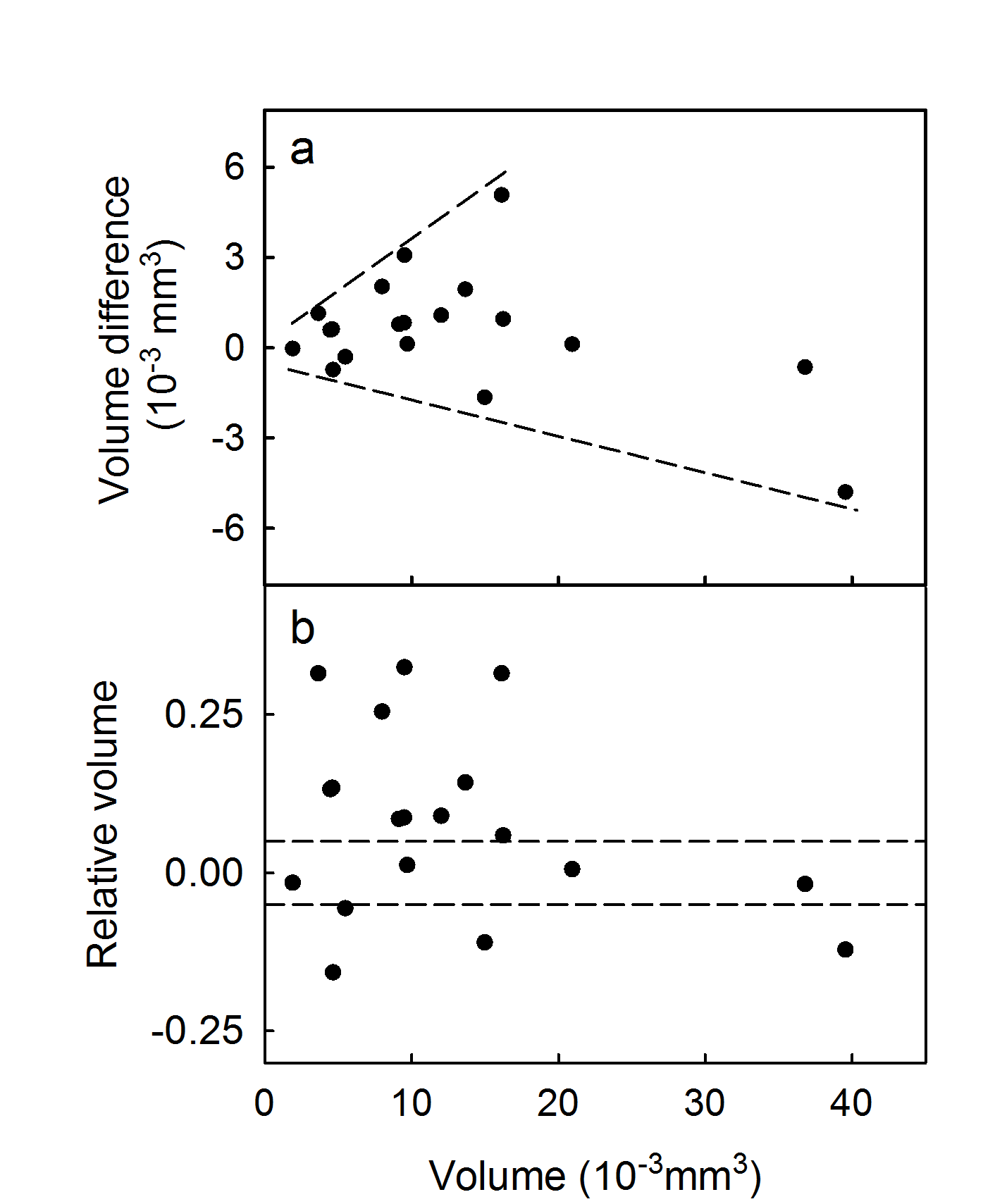

Supplement: Supplementary file 2 — Supplementary Figure 1 [file 41438_2020_274_MOESM2_ESM.tif]
